# Simultaneous Hybrid Treatment of Multilevel Peripheral Arterial Disease in Patients with Chronic Limb-Threatening Ischemia

**DOI:** 10.3390/jcm10132865

**Published:** 2021-06-28

**Authors:** Felice Pecoraro, David Pakeliani, Salvatore Bruno, Ettore Dinoto, Francesca Ferlito, Domenico Mirabella, Mario Lachat, Bianca Cudia, Guido Bajardi

**Affiliations:** 1 Department of Surgical, Oncological and Oral Sciences, University of Palermo, 90127 Palermo, Italy; salvatorebruno93@gmail.com (S.B.); francescaferlito.ff@gmail.com (F.F.); bianca.cudia@unipa.it (B.C.); guido.bajardi@unipa.it (G.B.); 2 Vascular Surgery Unit, AOUP Policlinico “P. Giaccone”, 90127 Palermo, Italy; ettoredinoto@gmail.com (E.D.); dmirabella@live.it (D.M.); 3 Vascular Surgery Unit, Ospedali Riuniti Villa Sofia-Cervello, 90100 Palermo, Italy; davidpakeliani@gmail.com; 4 Aortic and Vascular Center Hirslanden, 8032 Zurich, Switzerland; mario.lachat@hotmail.com

**Keywords:** hybrid treatment, endovascular treatment, simultaneous, critical limb ischemia, peripheral arterial disease

## Abstract

Background: Hybrid treatments (HT) aim to reduce conventional open surgery invasiveness and address multilevel peripheral arterial disease (PAD). Herein, the simultaneous HT treatment in patients with chronic limb-threatening ischemia (CLTI) is reported. Methods: Retrospective analysis, for the period from May 2012 to April 2018, of patients presenting multilevel PAD with CLTI addressed with simultaneous HT. The outcomes of these interventions were measured the following metrics: early technical successes (within 30 days following treatment) and late technical successes (30 days or more following treatment) and included mortality, morbidity symptoms recurrence, and amputation. Survival and patencies were estimated. The median follow-up was 43.77 months. Results: In the 45 included patients, the HT consisted of femoral bifurcation patch angioplasty followed by an endovascular treatment in 38 patients (84.4%) and endovascular treatment followed by a surgical bypass in 7 patients (15.6%). Technical success was 100% without perioperative mortality. Eight (17.8%) patients presented early complications without major amputations. During the follow-up, seven (15.6%) deaths occurred and six patients (13.3%) experienced symptoms recurrence, with five of those patients requiring major amputation. An estimated survival time of 5 years, primary patency, and secondary patency was 84.4%, 79.2%, and 83.3% respectively. Conclusions: Hybrid treatments are effective in addressing patients presenting with multilevel PAD and CLTI. The common femoral artery involvement influences strategy selection. Larger studies with longer-term outcomes are required to validate the hybrid approach, indications, and results.

## 1. Introduction

Lower limb peripheral arterial disease (PAD) is a continuously increasing disease with an estimated incidence of 500/million new cases yearly. PAD is associated with a relevant risk of limb loss when presenting at advanced stages as chronic limb-threatening ischemia (CLTI) [1,2]. In these patients, endovascular treatment (EVT) replaced conventional open surgery in most cases because of reduced mortality and morbidity rates despite a higher risk of occlusion [3,4].

Limitations of EVT are multilevel PAD distribution and femoral artery bifurcation involvement [5]. The hybrid treatment (HT) for PAD, first described in 1973 [6], combines both endovascular and surgical procedures simultaneously or in staged operations.

High-risk surgical patients benefit from a less invasive HT, allowing for multilevel PAD treatment and the reduction of the risks associated with conventional open surgery [7].

Herein we report our experience with simultaneous HT in patients with multilevel PAD presenting CLTI.

## 2. Materials and Methods

**Figure 1:**
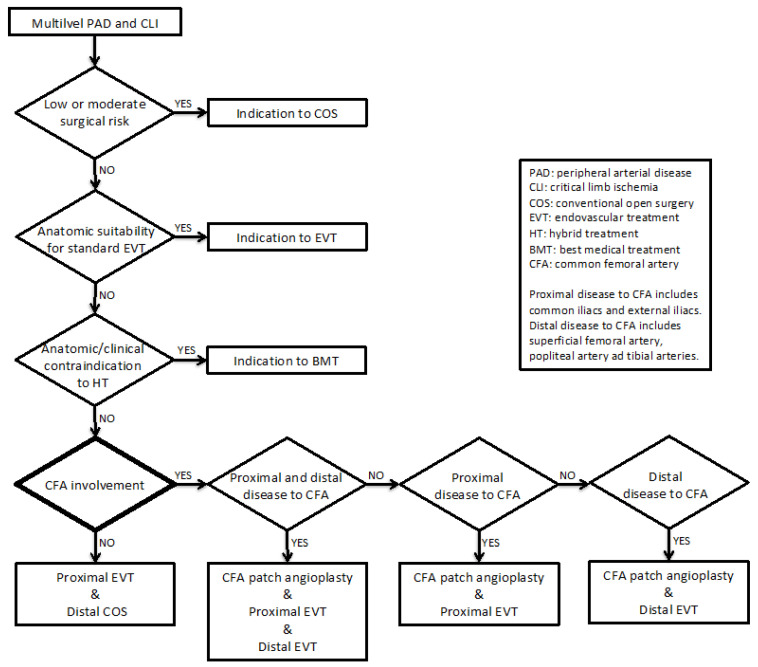
Flow-chat showing the decisional algorithm employed in patients presenting chronic limb-threatening ischemia with multilevel peripheral arterial disease.

From May 2012 to April 2018, consecutive data of all patients presenting with multilevel PAD with CLTI managed by HT were collected and inserted into standardized piloted forms. Indications of HTs were (i) multilevel PAD and CLTI, (ii) high risk for conventional open surgery, and (iii) anatomic unsuitability for standard EVT (Figure 1).

The CLTI criteria were rest pain (Rutherford category 4–6) [8], non-healing ulceration, or gangrene with an ankle-brachial index (ABI) < 0.39 [1]. CLTI patient classification was done according to the Society for Vascular Surgery Lower Extremity Threatened Limb Classification System: Risk Stratification Based on Wound, Ischemia, and foot Infection (WIfI) [9]. Only patients who were presenting CLTI and multilevel lesions being addressed with simultaneous HT were included. The exclusion criteria were: acute limb ischemia, nonatherosclerotic chronic vascular conditions of the lower extremity, and staged HTs. A multilevel reconstruction included treatment involving at least two arterial segments (aortoiliac; common femoral artery (CFA); deep femoral artery (DFA); superficial femoral artery (SFA); popliteal artery; or tibial arteries). The Trans-Atlantic Inter-Society Consensus II (TASC II) was employed to classify arteries addressed by ET for both aorto-iliac and femoro-popliteal disease [1]. The preoperative assessment consisted of a duplex ultrasound (DUS) in all cases. Computed tomography (CT) angiography was employed in patients presenting aorto-iliac lesions.

The collected variables were demographics, comorbidities, clinical data, preoperative imaging studies, procedure details, type of intervention, type of anesthesia, blood transfusions, medical therapy, and length of stay. Renal function was estimated with the Chronic Kidney Disease Epidemiology Collaboration (CKD-EPI) [10].

The collected data were retrospectively analyzed in December 2020. All patients gave informed consent for the procedure itself and for anonymous data collection and analysis.

The measured metrics included early technical successes (within 30 days following treatment) and late technical successes (30 days or more following treatment). A patent vessel with <30% residual stenosis following surgical reconstruction and EVT post-dilatation was the definition of technical success. Early outcomes measured included in-hospital mortality, morbidity, symptom recurrence, and amputation (major and minor). Major amputation was defined as any amputation performed above the level of the ankle, and minor amputation was defined as any amputation at level of or below the ankle. Late outcomes included mortality, symptoms recurrence, amputation (major and minor), survival, primary patency, and secondary patency. Loss of patency was calculated on a patient basis and was defined as thrombosis and/or occlusion of any treated vessel.

Correlation analysis of age, comorbidities, type of treatment, blood transfusion, reinterventions, a hospital stay with complications, amputation rate, and death was performed.

Follow-up consisted of a clinical examination and DUS at 1 week; after 3, 6, and 12 weeks; and every 6 months thereafter. The median follow-up was 35.57 (mean: 36.92; r: 1–90; standard deviation [SD]: 20) months.

For statistical analysis, means and SD or median and range were reported for parametric data; absolute values and percentages were reported for non-parametric data. Differences in preoperative and postoperative outcomes were assessed using the Student *t* test. Kaplan-Meier curves were used to estimate survival, primary patency, and secondary patency. A bivariate test was used to assess relationship significance for correlation analysis.

Statistical significance was considered at *p* < 0.05. For the Kaplan-Meier curves, a standard error exceeding 10% was reported. Statistical analysis was performed using SPSS 16.0 (SPSS Inc., Chicago, IL, USA). STROBE guidelines for reporting observational studies were followed [11].

**Figure 2:**
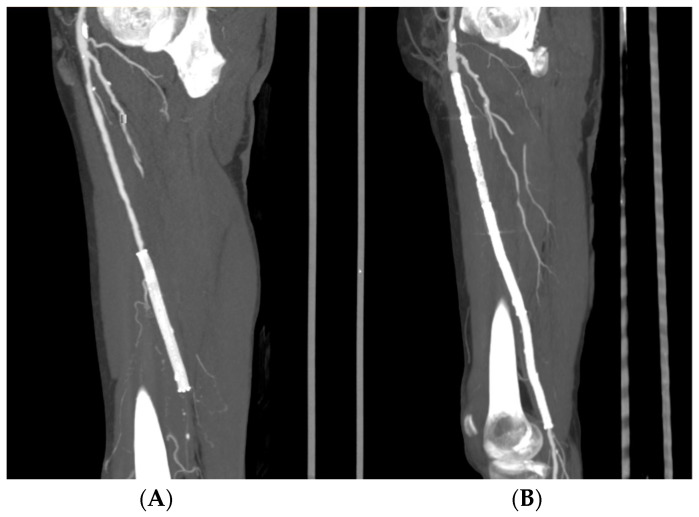
Preoperative CT MPR showing multilevel peripheral arterial disease compared to a common superficial artery, an in stent occlusion, and a distal superficial femoral artery occlusion (**A**). Postoperative CT MPR showing the outcome after common femoral artery patch angioplasty, stent recanalization, and superficial femoral artery stenting (**B**).

**Technique.** Vascular surgeons performed all of the hybrid procedures in a standard operating room equipped with a mobile C-arm system. HT for multilevel peripheral arterial disease involving the CFA consisted of endarterectomy and patch angioplasty, followed by EVT through the femoral patch. Patients without CFA involvement underwent a combination of EVT followed by a surgical bypass (Figure 2A,B).

### 2.1. HT with Femoral Artery Patch Angioplasty and Endovascular Treatment (HT-Patch)

The first step consisted of an endarterectomy and patch to maintain or restore adequate DFA outflow.

Upon completion of patch angioplasty and flow restoration, a 5–0 Prolene surgiclose ‘Z’ shape stitch was placed on the patch graft, as described elsewhere [12]. The EVT began with the femoral patch puncture using an 18G needle in correspondence to the surgiclose stitch.

Puncture direction was antegrade to address femoro-popliteal-tibial lesions and retrograde for aorto-iliac lesions. To facilitate the patch puncture, the needle was bent, maintaining a Teflon-coated guide inside. Subsequently, a 6F or 7F introducer sheath was advanced in the chosen direction in a standard fashion.

**Figure 3:**
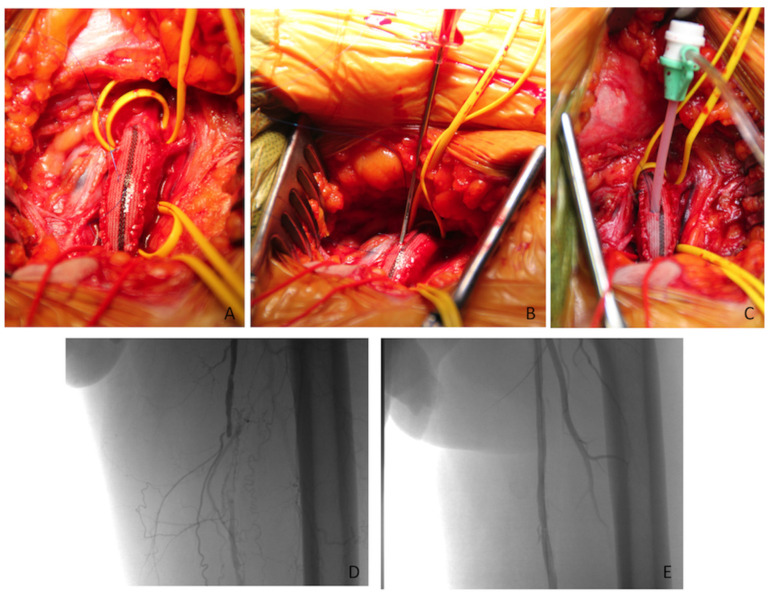
Intraoperative imaging showing (**A**) surgiclose placement in correspondence to a femoral dacron patch; (**B**) patch puncture using a bent 18G needle in antegrade direction; (**C**) placement of a 6F introducerin antegrade direction. (**D**) Angiography from the introducer through the patch positioned into the common femoral artery showing superficial femoral artery occlusion; (**E**) final angiographic result in correspondence of the superficial femoral artery.

The EVT was carried out based on lesion localization, extension, and preliminary angiography findings. Upon completion of the EVT, the endovascular materials were removed; the ‘Z’ stitch was tightened and knotted (Figure 3A–E).

**Figure 4:**
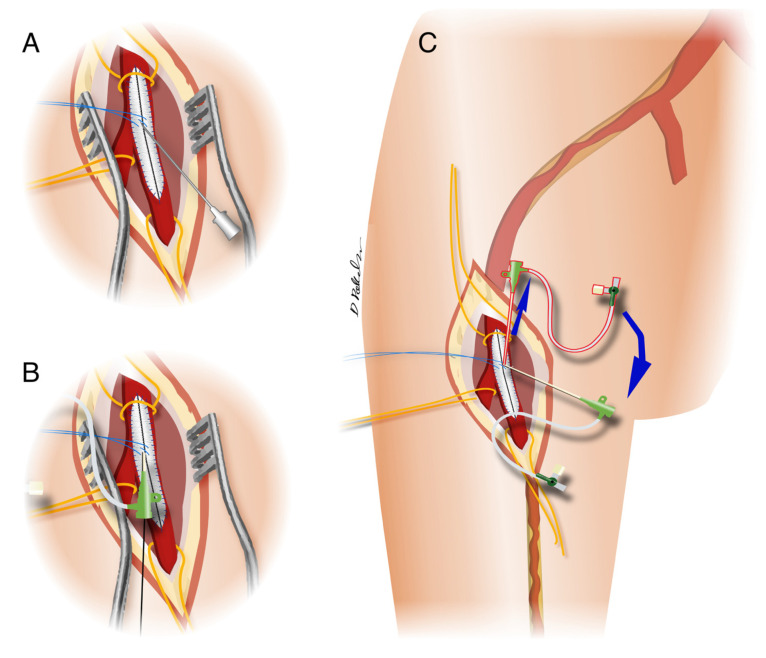
Drawings showing (**A**) retrograde puncture through the femoral patch in correspondence to the surgiclose “Z” shape stitch; (**B**) over the wire retrograde introducer placing to address iliac lesions; (**C**) surgiclose ‘Z’ stitch tightening with introducer removal and patch hole that was re-addressed using the introducer tip orientated in the antegrade fashion.

With a simple trick, lesions localized in the iliacs and below the CFA were simultaneously addressed by endovascular treatment: at iliac lesion treatment completion, the sheath was withdrawn, the ‘Z’ stitch was tightened but left unknotted, and the patch hole re-addressed using the introducer tip orientated in the antegrade direction to address the distal CFA lesions (Figure 4A–C).

### 2.2. HT with Endovascular Treatment and Bypass Surgery (HT-Bypass)

The first step consisted of the surgical exposure of the femoral bifurcation. A 5–0 Prolene surgiclose ‘Z’ shape stitch was placed [12] in correspondence to the CFA. A direct arterial puncture was performed in the retrograde direction in the middle of the surgiclose stitch and the EVT was performed. At the end of the EVT, the arterial puncture was closed using the surgiclose stitch. Thereafter, surgery was performed traditionally with a femoro-popliteal bypass above or below the knee according to the location of the lesion.

Iliac disease was managed with EVT; the SFA lesions were able to be managed with EVT or COS, according to the disease extension [1] and patient comorbidities.

The endovascular treatment for aorto-iliac lesions consisted of stents or stent-graft placement; for SFA lesions a drug-eluting-balloon (DEB), stenting, or standard transluminal angioplasty were used for treatment; for infragenicular lesions, treatment was possible with DEB or standard ballooning. In all cases, a complete angiography was carried out to assess both technical success and residual stenosis. A general heparinization, with an activated clotting time >180 s, was administered to perform all of the HTs.

## 3. Results

**Patient characteristics.** The current study included 45 patients with a mean age of 71 (r: 49–88; SD: 10) years. During the study period, an increase of HTs was observed (11 cases May 2012–April 2015 vs. 34 cases May 2015–April 2018; *p* < 0.001). Comorbidities and preoperative findings are summarized in Table 1. According to the Rutherford classification, 15 (33.3%) patients were in category 4; 21 (46.7%) in Rutherford category 5; and 9 (20%) in Rutherford category 6. Based on the WIfI risk stratification system, 13 (28.9%) patients were categorized into grade 4; 29 (64.4%) into grade 3; and the remaining 3 (6.7%) into grade 2.

Contemporary multilevel lesions were encountered at 2 levels in 19 (42.2%) patients; at 3 levels in 13 (28.9%) patients; at 4 levels in 9 (20%) patients; at 5 levels in 2 (4.4%) patients; and at 6 levels in 2 (4.4%) patients (Table 1). The mean preoperative ABI of the target lower extremity was 0.32 (r:0.21–0.44; SD: 0.22). Before HT, 40 patients received antiplatelet or anticoagulant therapy as cardioaspirin (23; 51.1%); clopidogrel (3; 6.7%); cardiaspirin and clopidogrel (11; 24.4%); and oral anticoagulants (3; 6.7%).

**Treatment.** The mean HT duration was 187 (r:90–400; SD 69) min, with 24 (53%) cases requiring ≥ 180 min. Twenty-five (55.6%) patients underwent HT under local anaesthesia; 11 (24.4%) under general anaesthesia; and 9 (20%) under spinal anaesthesia.

The HT-Patch was used in 38 (84.4%) patients and HT-Bypass was used in in 7 (15.6%) patients. All femoral bifurcation patch angioplasties were performed using a Dacron patch graft (Vascutek, Terumo Company, Inchinnan, UK). Surgical bypasses above the knee were carried out in five cases with a reinforced 8 mm Dacron (3) and PTFE (2) (Vascutek, Terumo Company). The remaining two bypasses were performed below the knee using a reversed great saphenous vein.

The EVTs were performed in antegrade fashion in 21 (46.7%) patients; in retrograde fashion for iliac arteries in 19 (42.2%) patients; and in both directions in 5 (11.1%) patients. All of the procedures requiring both antegrade and retrograde directions were carried through a femoral Dacron patch (Table 2).

A significant increase in postoperative ABI was reported after the HTs [0.32 (r:0.21–0.44; SD: 0.22) vs. 0.77 (r:0.63–1, SD: 0.31); *p* = 0.003].

ICU stay was required in 12 (26.7%) patients after the HT, with a mean length of stay of 1.8 (r:1–5; SD:1) days. The mean hospital stay was 9 (r: 1–33; SD: 6) days. No significant differences were observed in GFR before and after the HT (60.1 ± 29.9 vs. 64.8 ± 30.7; *p* = 0.08). Five (11.1%) patients required blood transfusion after the intervention.

At discharge, antiplatelet and/or oral anticoagulants were administered to all patients, including cardioaspirin and clopidogrel to 36 (80%) patients; cardioaspirin to 8 (17.8%) patients; and cardioaspirin with oral anticoagulants to 1 (2.2%) patient.

**Early Outcomes.** Technical success was achieved in all patients. No in-hospital mortality was registered. Perioperative morbidity was observed in four (6.7%) patients and consisted of bleeding from a ruptured EIA requiring reintervention (one); non-fatal myocardial infarction (one); inguinal lymphatic fistula (one); and acute renal failure requiring 2 days transient dialysis (one). Early symptom recurrence occurred in two patients presenting SFA uncovered stent occlusion (one) and reinforced Dacron AK bypass occlusion (one). These patients were addressed respectively with an additional PTA and stent distal extension and mechanical embolectomy with local fibrinolysis within 24 h from the symptom recurrence onset for the occluded bypass graft. Perioperative minor amputations were necessary for two (4.4%) patients.

**Late outcomes.** During the median follow-up of 43.77 (mean: 45.11; r: 1–98; SD: 22) months, seven (15.6%) deaths were registered. In two cases, death occurred within 90 days of intervention and was related to myocardial infarction in one case (31th POD) and acute renal insufficiency in the other (38th POD). In the remaining patients, death was secondary to cardiac disease (two); cancer (two); and unknown causes (one).

**Figure 5:**
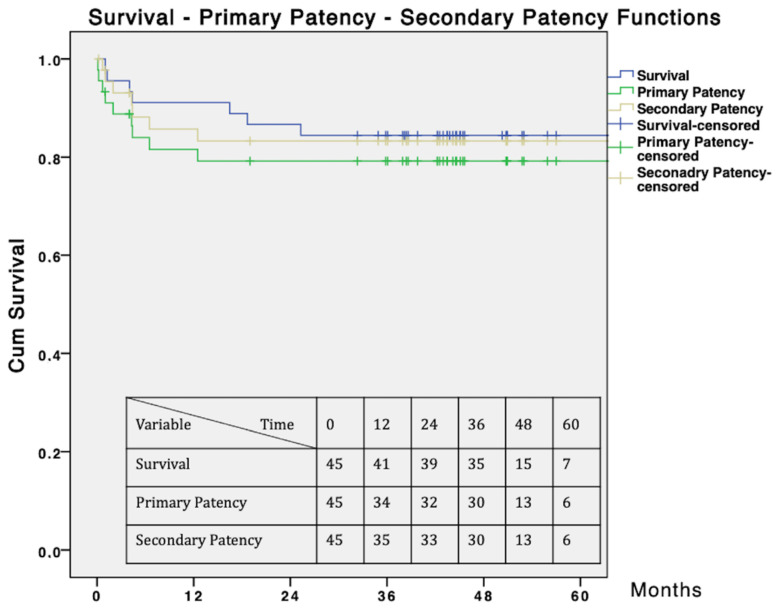
Survival, primary patency, and secondary patency curves. Standard error does not exceed 10% at 48 months for the survival, primary patency, and secondary patency curves.

Late symptom recurrence occurred in six (13.3%) patients; of these, five underwent major amputation, and the remaining patient was able to receive another SFA stenting. During the follow-up, one (2.2%) minor amputation was required. The estimated 60-month survival time, primary patency, and secondary patency were 84.4%, 79.2%, and 83.3% respectively (Figure 5).

The Pearson correlation coefficient showed a strong correlation of the amputation rate with the history of PCI or CABG (*p* = 0.003), postoperative complications (*p* < 0.001), and the number of arterial segments involved (*p* = 0.009); a longer hospital stay was associated with higher long-term mortality rates (*p* = 0.009). The full correlation analysis is shown in Table 3.

## 4. Discussion

Patients affected by CLTI present a multilevel arterial involvement of up to 90% [13]. To achieve consistent clinical improvements, these patients require extensive multilevel reconstruction [13,14,15]. Conversely, limited arterial reconstructions are often inadequate to ensure limb survival [13,16].

The first-line option for patients presenting multilevel PAD is still conventional open surgery. These invasive procedures are associated with a high incidence of mortality and morbidity, up to 8% and 24% in the current literature [17]. Moreover, it is not feasible in up to 40% of cases due to the association with significant patient co-morbidities and poor life expectancy [18].

Total endovascular solutions have been reported as a valuable option in such frail patients to reduce surgical invasiveness [14].

However, multilevel endovascular solutions represent an independent risk factor for failure and primary patency [19]. A recent meta-analysis, including patients with and without multilevel PAD, reported a rate of perioperative mortality of 1–7%; perioperative complications at a rate of 1–7%; and major amputation at a rate of 0–7% after EVT [20].

In the investigated population, the mortality after 1 year is extremely low when compared to the normal or CLTI populations [1,3]. In addition, the major amputation rate was lower after 1 year when compared to literature, specifically25% [2,8].

Additionally, the involvement of the common femoral artery and its bifurcation is a limitation to endovascular treatments [21]. In fact, ESC guidelines recommend against the use of endovascular treatments for CFA disease [22] despite the recent literature reports indicating acceptable results of this approach [23].

The involvement of the common femoral artery in multilevel CLTI is infrequent, and the usual disease localization is in the femoro-popliteal and distal districts.

ESC guidelines advocate the CFA involvement as a significant determinant for the treatment choice algorithm in both aortoiliac and femoropopliteal lesions [22].

Despite there being no clear indication in how to address aorto-iliac and femoro-popliteal lesions simultaneously, in our experience, the common femoral artery with its bifurcation is a determinant in treatment strategy selection (Figure 1).

HT has been introduced to increase the number of treated vessels and reduce surgical invasiveness by combining open and endovascular techniques [19]. These HTs are continuously increasing to include up to 20% of peripheral vascular interventions [24].

However, HT procedures are reserved for patients presenting CLTI that requires extensive revascularization to reduce invasiveness due to their frail condition [25,26,27,28]. This study included only patients with CLTI requiring multilevel revascularization, who were considered unfit for open surgery or endovascular treatments. In these patients, arterial lesions were treated with two types of procedures:

- Femoral artery patch angioplasty and endovascular treatment and HT-Patch;
- HT with endovascular treatment and bypass surgery HT-Bypass.

The HT-Patch was performed in patients presenting CFA with atherosclerotic involvement; in such cases, the first step consisted of a surgical femoral Dacron patch carried out with limited surgical exposure. It allowed the subsequent remote antegrade and/or retrograde arterial recanalization using EVT.

It has been reported by Dosluoglu et al. [7] that a key maneuver when performing patch angioplasty and subsequent endovascular treatment is to achieve an inflow or outflow guidewire access before performing the arteriotomy. In our experience, successful guidewire access was obtained through the patch graft in all patients after the CFA patch angioplasty during the HT-patch interventions. This maneuver i) avoids the manipulation of a fragile arterial wall during the introducer sheath placement; ii) limits the clamping time of the patch placement; and iii) allows the performance of the endovascular operation with the CFA unclamped.

The HT-bypass was indicated in patients with no CFA atherosclerotic involvement; in such cases, the CFA was employed to endovascularly address the proximal arterial lesions in correspondence to the aorto-iliac segment; subsequently, the exposed CFA was the inflow site for the surgical bypasses. In these patients managed by HT-bypass, a fully endovascular intervention was not attempted because of a diseased contralateral iliac-femoral axis, lesions involving the base of the common iliac artery, a diseased aorta obstructing brachial access, and/or disease of the SFA artery involving the ostium. Moreover, a long (>20 cm) diseased SFA was diagnosed.

As reported, CFA involvement was determinant for the choice of HT approach (HT-patch or HT-bypass).

Despite a long operative time (at least 180 min) in 53% of cases, most of these procedures were carried out under local or spinal anesthesia to reduce the risks related to general anesthesia.

HTs can be performed simultaneously or staggered depending on whether the open procedures and the endovascular procedures are performed in the same operation or delayed.

The decision to perform staggered vs. combined inflow and outflow revascularization should be based on patient risk and the severity threat to the limb [27].

Reported advantages of simultaneous HTs are (a) the absence of delay in full revascularization; (b) inadequate results of both the COS and EVT can be corrected using these complementary techniques; (c) an endovascular access puncture can be performed under the direct control of a surgically exposed vessel; (d) a single operation exposes to single risk of infection; (e) there is no need for therapy adjustments between the staggered procedures; (f) a single operation reduces the length of the hospital stay; and (g) the use of intraoperative angiography allows also the routine control of surgical procedures [28].

Simultaneous hybrid procedures may significantly reduce hospital charges and length of stay, as reported by Ebaugh et al. It was observed that the costs of staggered hybrid treatments are doubled when compared to simultaneous treatments [24].

In reported experiences, early occlusions were addressed with successful reintervention and associated with being able to salvage the limb. Conversely, in all but one case, late occlusions were associated with major amputation. Additionally, in the newly released global vascular guidelines for the management of chronic limb-threatening ischemia, there is scarce information about HT application, and what is available lacks strong evidence.

Hopefully, this study could contribute to the future applicability of HT by demonstrating its safety and its validity as an alternative to the exclusively endovascular or surgical approach [27].

Study limitations are the retrospective single-centre analysis, the lack of a control group, and the limited follow-up period. Another limitation is the variable pattern of arterial disease and the use of two different treatment strategies.

## 5. Conclusions

In the reported experiences, simultaneous hybrid treatments of multilevel peripheral arterial disease in high-risk patients was feasible with good outcomes. Common femoral artery involvement plays a determinant role in strategy selection. Early occlusion was associated with lower limb-loss risk when compared to late occlusions. A higher risk of amputation occurred in patients presenting a history of previous cardiac interventions and a larger amount of arterial segment involvement. A longer hospital stay was associated with higher long-term mortality rates.

## Figures and Tables

**Table 1:** Comorbidities and preoperative findings.

	No. (%)
Patients	45
Male	36 (80)
Hypertension	39 (86.7)
Diabetes	23 (51.1)
Cardiac Disease	23 (51.1)
Previous PCI/CABG	18 (40)
Cancer	7 (15.6)
Lipid disorders	33 (73.3)
COPD	35 (77.8)
GFR < 60	21 (46.7)
Dialysis	4 (8.9)
Previous PAD intervention	18 (40)
Endovascular	14 (31.1)
Open	4 (8.9)
ASA	
III	27 (60)
IV	18 (40)
Previous vascular surgery	4 (8.9)
Previous endovascular surgery	14 (31.1)
Clinical presentation	
Rutherford IV	15 (33.3)
Rutherford V	21 (46.7)
Rutherford VI	9 (20)
Diseased vessels	
Iliac	26 (57.8)
Common femoral artery	30 (66.7)
Deep femoral artery	12 (26.7)
Superficial femoral artery	39 (86.7)
Popliteal	11 (24.4)
Tibial	17 (37.8)
TASC II	
A	7 (15.6)
B	11 (24.4)
C	15 (33.3)
D	12 (26.7)

No: number; PCI/CABG: percutaneous coronary intervention/coronary artery bypass graft; COPD: chronic obstructive pulmonary disease; GFR: glomerular filtration rate; PAD: peripheral arterial disease; TASC: Trans-Atlantic consensus society; ASA: American Society of Anesthesiologists.

**Table 2:** Hybrid treatment operative detail.

		No. (%)
Conventional open surgery		
Femoral patch	Dacron	38(84.4)
Bypasses AK	Dacron (reinforced)	3 (6.8)
	PTFE (reinforced)	2 (4.4)
Bypasses BK	GSV	2 (4.4)
Endovascular treatment		
Iliac Stenting	Covered	16 (35.6)
	Uncovered	9 (20)
SFA stenting	Uncovered	20 (44.4)
SFA PTA	DEB	6 (13.3)
	Standard	1 (2.2)
Popliteal PTA	DEB	6 (13.3)
Tibial	DEB	6 (13.3
	Standard	6 (13.3)

No: number; AK: above the knee; BK: below the knee; SFA: superficial femoral artery; PTA: percutaneous transluminal angioplasty; DEB: drug eluting balloon; GSV: great saphenous vein.

**Table 3:** Pearson Correlation.

		DM	PCI/CABG	CKD	Smoker	Seg	Bypass	Stenting	BT	Patch	Age	InH LOS	HT	30D Reint	Amput	Compl	Death
DM	Correlation		0.163	0.409 **	−0.222	0.041	−0.071	−0.174	−0.22	0.071	−0.093	0.305 *	0.139	0.174	−0.079	0.081	0.174
	Sig. (2-t)		0.284	0.005	0.142	0.791	0.644	0.252	0.146	0.644	0.544	0.041	0.361	0.252	0.608	0.596	0.252
PCI/ CABG	Correlation	0.163		0.096	−0.214	0.124	0.275	0.1	−0.144	−0.275	0.097	−0.017	−0.08	0.15	0.433 **	0.137	0.15
	Sig. (2-t)	0.284		0.529	0.159	0.416	0.067	0.513	0.344	0.067	0.528	0.913	0.601	0.325	0.003	0.369	0.325
CKD	Correlation	0.409 **	0.096		−0.411 **	0.344 *	−0.173	−0.217	−0.1	0.173	0.028	0.252	0	−0.043	0.05	0.238	0.347 *
	Sig. (2-t)	0.005	0.529		0.005	0.021	0.255	0.153	0.513	0.255	0.856	0.094	1	0.777	0.744	0.116	0.02
Smokers	Correlation	−0.222	−0.214	−0.411 **		−0.053	0.039	0.121	0.164	−0.039	−0.026	−0.313 *	0.16	−0.121	−0.021	−0.19	−0.442 **
	Sig. (2-t)	0.142	0.159	0.005		0.729	0.798	0.428	0.281	0.798	0.865	0.037	0.295	0.428	0.893	0.212	0.002
Seg	Correlation	0.041	0.124	0.344 *	−0.053		−0.224	0	−0.065	0.224	−0.055	0.291	−0.239	−0.168	0.387 **	0.263	0.28
	Sig. (2-t)	0.791	0.416	0.021	0.729		0.139	1	0.674	0.139	0.72	0.052	0.114	0.27	0.009	0.081	0.063
Bypass	Correlation	−0.071	0.275	−0.173	0.039	−0.224		−0.154	0.043	−1.00 **	−0.017	0.065	0.168	0.323 *	0.043	−0.156	−0.015
	Sig. (2-t)	0.644	0.067	0.255	0.798	0.139		0.312	0.777	0	0.913	0.672	0.269	0.03	0.777	0.306	0.922
Stenting	Correlation	−0.174	0.1	−0.217	0.121	0	−0.154		−0.043	0.154	−0.13	−0.103	−0.168	0.015	−0.043	−0.109	−0.323 *
	Sig. (2-t)	0.252	0.513	0.153	0.428	1	0.312		0.777	0.312	0.393	0.502	0.269	0.922	0.777	0.476	0.03
BT	Correlation	−0.22	−0.144	−0.1	0.164	−0.065	0.043	−0.043		−0.043	0.1	−0.078	0.139	0.043	−0.125	−0.085	−0.152
	Sig. (2-t)	0.146	0.344	0.513	0.281	0.674	0.777	0.777		0.777	0.513	0.612	0.364	0.777	0.413	0.579	0.32
Patch	Correlation	0.071	−0.275	0.173	−0.039	0.224	−1.00 **	0.154	−0.043		0.017	−0.065	−0.168	−0.323 *	−0.043	0.156	0.015
	Sig. (2-t)	0.644	0.067	0.255	0.798	0.139	0	0.312	0.777		0.913	0.672	0.269	0.03	0.777	0.306	0.922
Age	Correlation	−0.093	0.097	0.028	−0.026	−0.055	−0.017	−0.13	0.1	0.017		−0.032	−0.058	−0.305 *	−0.033	−0.106	0.079
	Sig. (2-t)	0.544	0.528	0.856	0.865	0.72	0.913	0.393	0.513	0.913		0.837	0.706	0.042	0.831	0.487	0.605
InH LOS	Correlation	0.305 *	−0.017	0.252	−0.313 *	0.291	0.065	−0.103	−0.078	−0.065	−0.032		−0.079	0.188	0.108	0.316 *	0.387 **
	Sig. (2-t)	0.041	0.913	0.094	0.037	0.052	0.672	0.502	0.612	0.672	0.837		0.604	0.216	0.48	0.034	0.009
HT	Correlation	0.139	−0.08	0	0.16	−0.239	0.168	−0.168	0.139	−0.168	−0.058	−0.079		−0.012	−0.277	−0.301 *	−0.192
	Sig. (2-t)	0.361	0.601	1	0.295	0.114	0.269	0.269	0.364	0.269	0.706	0.604		0.938	0.065	0.044	0.205
30D Reint	Correlation	0.174	0.15	−0.043	−0.121	−0.168	0.323 *	0.015	0.043	−0.323 *	−0.305 *	0.188	−0.012		−0.152	−0.156	−0.015
	Sig. (2-t)	0.252	0.325	0.777	0.428	0.27	0.03	0.922	0.777	0.03	0.042	0.216	0.938		0.32	0.306	0.922
Amput	Correlation	−0.079	0.433 **	0.05	−0.021	0.387 **	0.043	−0.043	−0.125	−0.043	−0.033	0.108	−0.277	−0.152		0.526 **	0.238
	Sig. (2-t)	0.608	0.003	0.744	0.893	0.009	0.777	0.777	0.413	0.777	0.831	0.48	0.065	0.32		0	0.115
Compl	Correlation	0.081	0.137	0.238	−0.19	0.263	−0.156	−0.109	−0.085	0.156	−0.106	0.316 *	−0.301 *	−0.156	0.526 **		0.109
	Sig. (2-t)	0.596	0.369	0.116	0.212	0.081	0.306	0.476	0.579	0.306	0.487	0.034	0.044	0.306	0		0.476
Death	Correlation	0.174	0.15	0.347 *	−0.442 **	0.28	−0.015	−0.323 *	−0.152	0.015	0.079	0.387 **	−0.192	−0.015	0.238	0.109	
	Sig0. (2-t)	0.252	0.325	0.02	0.002	0.063	0.922	0.03	0.32	0.922	0.605	0.009	0.205	0.922	0.115	0.476	

Orange: Strong positive correlation; Yellow: positive correlation; Green: negative correlation; **. Correlation is significant at the 0.01 level (2-tailed); *. Correlation is significant at the 0.05 level (2-tailed); DM: diabetes mellitus; PCI/CABG: percutaneous coronary intervention; CKD: chronic kidney disease; Seg: arterial segments; BT: blood transfusion; InH LOS: in hospital length of stay; HT: hypertension; 30D Reint: intervention at 30 days; Amput: amputation; Compl: complications; Sig. (2-t): significance (2-tailed).

## References

[1] Norgren L., Hiatt W.R., Dormandy J.A., Nehler M.R., Harris K.A., Fowkes F.G.R. (2007). Inter-society consensus for the management of peripheral arterial disease (TASC II). J. Vasc. Surg..

[2] Bracale U.M., Ammollo R.P., Hussein E.A., Hoballah J.J., Goeau-Brissonniere O., Taurino M., Setacci C., Pecoraro F., Bracale G., Giribono A.M. (2020). Managing peripheral artery disease in diabetic patients: A questionnaire survey from vascular centers of the mediterranean federation for the advancing of vascular surgery (MeFAVS). Ann. Vasc. Surg..

[3] Goodney P.P., Beck A.W., Nagle J., Welch H.G., Zwolak R.M. (2009). National trends in lower extremity bypass surgery, endovascular interventions, and major amputations. J. Vasc. Surg..

[4] Dinoto E., Pecoraro F., Mirabella D., Ferlito F., Farina A., lo Biundo N., Orlando-Conti P., Bajardi G. (2020). A single-center experience on below-the-knee endovascular treatment in diabetic patients. Transl. Med. UniSa.

[5] Thukkani A.K., Kinlay S. (2015). Endovascular intervention for peripheral artery disease. Circ. Res..

[6] Porter J.M., Eidemiller L.R., Dotter C.T., Rösch J., Vetto R.M. (1973). Combined arterial dilatation and femorofemoral bypass for limb salvage. Surg. Gynecol. Obstet..

[7] Dosluoglu H.H., Lall P., Cherr G.S., Harris L.M., Dryjski M.L. (2010). Role of simple and complex hybrid revascularization procedures for symptomatic lower extremity occlusive disease. J. Vasc. Surg..

[8] Rutherford R.B., Baker J.D., Ernst C., Johnston K.W., Porter J.M., Ahn S., Jones D.N. (1997). Recommended standards for reports dealing with lower extremity ischemia: Revised version. J. Vasc. Surg..

[9] Mills J.L., Conte M.S., Armstrong D.G., Pomposelli F.B., Schanzer A., Sidawy A.N., Andros G., on behalf of the Society for Vascular Surgery Lower Extremity Guidelines Committee (2014). The society for vascular surgery lower extremity threatened limb classification system: Risk stratification based on wound, ischemia, and foot infection (WIfI). J. Vasc. Surg..

[10] Levey A.S., Stevens L.A., Schmid C.H., Zhang Y., Castro A.F., Feldman H.I., Kusek J.W., Eggers P., van Lente F., Greene T. (2009). A new equation to estimate glomerular filtration rate. Ann. Intern. Med..

[11] Von Elm E., Altman D.G., Egger M., Pocock S.J., Gøtzsche P.C., Vandenbroucke J.P. (2007). The strengthening the reporting of observational studies in epidemiology (STROBE) statement: Guidelines for reporting observational studies. Lancet Lond. Engl..

[12] Mayer D., Rancic Z., Wilhelm M., Genoni M., Veith F.J., Lachat M. (2008). Improved hybrid technique for vascular access and closure. J. Endovasc. Ther. Off. J. Int. Soc. Endovasc. Spec..

[13] Ghoneim B., Elwan H., Eldaly W., Khairy H., Taha A., Gad A. (2014). Management of critical lower limb ischemia in endovascular era: Experience from 511 patients. Int. J. Angiol. Off. Publ. Int. Coll. Angiol. Inc..

[14] Kinlay S. (2016). Management of critical limb ischemia. Circ. Cardiovasc. Interv..

[15] Bracale U.M., Vitale G., Bajardi G., Narese D., Dinoto E., Giribono A.M., Ferrara D., del Guercio L., Midiri M., Pecoraro F. (2016). Use of the directional atherectomy for the treatment of femoro-popliteal lesions in patients with critical lower limb ischemia. Transl. Med. UniSa.

[16] Blevins W.A., Schneider P.A. (2010). Endovascular management of critical limb ischemia. Eur. J. Vasc. Endovasc. Surg. Off. J. Eur. Soc. Vasc. Surg..

[17] Farber A., Eberhardt R.T. (2016). The current state of critical limb ischemia: A systematic review. JAMA Surg..

[18] Soga Y., Iida O., Takahara M., Takahaera M., Hirano K., Suzuki K., Kawasaki D., Miyashita Y., Tsuchiya T. (2014). Two-year life expectancy in patients with critical limb ischemia. JACC Cardiovasc. Interv..

[19] Antoniou G.A., Sfyroeras G.S., Karathanos C., Achouhan H., Koutsias S., Vretzakis G., Giannoukas A.D. (2009). Hybrid endovascular and open treatment of severe multilevel lower extremity arterial disease. Eur. J. Vasc. Endovasc. Surg. Off. J. Eur. Soc. Vasc. Surg..

[20] Almasri J., Adusumalli J., Asi N., Lakis S., Alsawas M., Prokop L.J., Bradbury A., Kolh P., Conte M.S., Murad M.H. (2018). A systematic review and meta-analysis of revascularization outcomes of infrainguinal chronic limb-threatening ischemia. J. Vasc. Surg..

[21] Drachman D.E., Armstrong E.J. (2017). Stenting the common femoral artery: Crossing the rubicon of endovascular treatment?. JACC Cardiovasc. Interv..

[22] Aboyans V., Ricco J.-B., Bartelink M.-L.E.L., Björck M., Brodmann M., Cohnert T., Collet J.-P., Czerny M., de Carlo M., Debus S. (2018). 2017 ESC guidelines on the diagnosis and treatment of peripheral arterial diseases, in collaboration with the European Society for Vascular Surgery (ESVS). Eur. Heart J..

[23] Siracuse J.J., van Orden K., Kalish J.A., Eslami M.H., Schermerhorn M.L., Patel V.I., Rybin D., Farber A. (2017). Endovascular treatment of the common femoral artery in the Vascular Quality Initiative. J. Vasc. Surg..

[24] Ebaugh J.L., Gagnon D., Owens C.D., Conte M.S., Raffetto J.D. (2008). Comparison of costs of staged versus simultaneous lower extremity arterial hybrid procedures. Am. J. Surg..

[25] Jones W.S., Mi X., Qualls L.G., Vemulapalli S., Peterson E.D., Patel M.R., Curtis L.H. (2015). Trends in settings for peripheral vascular intervention and the effect of changes in the outpatient prospective payment system. J. Am. Coll. Cardiol..

[26] Pecoraro F., Dinoto E., Bracale U.M., Badalamenti G., Farina A., Bajardi G. (2017). Symptomatic deep femoral artery pseudoaneurysm endovascular exclusion. Case report and literature review. Ann. Vasc. Surg..

[27] Pecoraro F., Bajardi G., Dinoto E., Vitale G., Bellisi M., Bracale U.M. (2015). Endograft connector technique to treat popliteal artery aneurysm in a morbid obese patient. Vascular.

[28] Dinoto E., Pecoraro F., Cutrupi A., Bracale U.M., Panagrosso M., Bajardi G. (2020). Single staged hybrid approach for multilevel aortic-iliac-femoral-popliteal disease. Int. J. Surg. Case Rep..

